# Nrf2 Lowers the Risk of Lung Injury via Modulating the Airway Innate Immune Response Induced by Diesel Exhaust in Mice

**DOI:** 10.3390/biomedicines8100443

**Published:** 2020-10-21

**Authors:** Ying-Ji Li, Takako Shimizu, Yusuke Shinkai, Tomomi Ihara, Masao Sugamata, Katsuhito Kato, Maiko Kobayashi, Yukiyo Hirata, Hirofumi Inagaki, Makoto Uzuki, Toshio Akimoto, Masakazu Umezawa, Ken Takeda, Arata Azuma, Masayuki Yamamoto, Tomoyuki Kawada

**Affiliations:** 1 Department of Hygiene and Public Health, Nippon Medical School, 1-25-16 Nezu, Bunkyo-ku, Tokyo 113-0031, Japan; takako-s@nms.ac.jp (T.S.); katzkato@nms.ac.jp (K.K.); mk831111@nms.ac.jp (M.K.); yuki-hir@nms.ac.jp (Y.H.); hrfmi@nms.ac.jp (H.I.); kawada@nms.ac.jp (T.K.); 2 Center for Environmental Health Science for the Next Generation, Research Institute for Science and Technology, Tokyo University of Science, Noda 278-8510, Japan; shinkai49@gmail.com (Y.S.); iharat@pure.ocn.ne.jp (T.I.); mspathol@beige.ocn.ne.jp (M.S.); masa-ume@rs.noda.tus.ac.jp (M.U.); takedak@rs.socu.ac.jp (K.T.); 3 Tochigi Institute of Clinical Pathology, Tochigi 329-0112, Japan; 4 Division of Laboratory Animal Science, Nippon Medical School, Tokyo 113-0031, Japan; doukan@nms.ac.jp (M.U.); toshio@nms.ac.jp (T.A.); 5 Department of Pulmonary Medicine and Oncology, Nippon Medical School, Tokyo 113-8602, Japan; azuma_arata@yahoo.co.jp; 6 Medical Biochemistry, Tohoku University Graduate School of Medicine, Sendai 980-8575, Japan; masiyamamoto@med.tohoku.ac.jp

**Keywords:** oxidative stress/anti-oxidative stress, immune response, macrophage, neutrophils, lung diseases

## Abstract

In the present study, we investigated the role of Nrf2 in airway immune responses induced by diesel exhaust (DE) inhalation in mice. C57BL/6J *Nrf2*^+/+^ and *Nrf2*^−/−^ mice were exposed to DE or clean air for 8 h/day and 6 days/week for 4 weeks. After DE exposure, the number of neutrophils and macrophage inflammatory protein (MIP)-2 level in bronchoalveolar lavage fluid (BALF) and interleukin (IL)-17 level in the lung tissue increased in *Nrf2*^−/−^ mice compared with *Nrf2*^+/+^ mice; however, the lack of an increase in the level of tumor necrosis factor (TNF)-α in the lung tissue in *Nrf2*^+/+^ mice and mild suppression of the level of TNF-α in *Nrf2*^−/−^ mice were observed; the level of granulocyte macrophage colony-stimulating factor (GM-CSF) in the lung tissue decreased in *Nrf2*^−/−^ mice than in *Nrf2*^+/+^ mice; the number of DE particle-laden alveolar macrophages in BALF were larger in *Nrf2*^−/−^ mice than in *Nrf2*^+/+^ mice. The results of electron microscope observations showed alveolar type II cell injury and degeneration of the lamellar body after DE exposure in *Nrf2*^−/−^ mice. Antioxidant enzyme NAD(P)H quinone dehydrogenase (NQO)1 mRNA expression level was higher in *Nrf2*^+/+^ mice than in *Nrf2*^−/−^ mice after DE exposure. Our results suggested that Nrf2 reduces the risk of pulmonary disease via modulating the airway innate immune response caused by DE in mice.

## 1. Introduction

The alveolar membrane is the largest surface of the body in contact with the outside environment. The lungs are continuously exposed to a diverse array of microbes and organic and inorganic particulate materials, and thus they are the organ most susceptible to air pollution. Epidemiological studies have shown that fine particulate matter of diameter ≤2.5 µm (PM2.5) is associated with increased respiratory morbidity and mortality [1,2]. PM2.5 includes primary particles that are emitted directly from sources such as fossil fuel combustion, for example, diesel exhaust (DE) particles (DEP), and secondary particles that are generated from gases through chemical reactions [3]. Many in vivo and in vitro studies have been performed to clarify the association between DEP and diseases, particularly pulmonary diseases [4,5]. DEP consists of a carbonaceous core with a large surface area, to which chemicals become adsorbed. These chemicals include organic chemicals, such as polycyclic aromatic hydrocarbons (PAHs), nitro derivatives of PAHs, and oxygenated derivatives of PAHs (ketones, quinones, and diones), heterocyclic compounds, aldehydes, and aliphatic hydrocarbons. PAHs and their oxygenated derivatives (e.g., quinones) have been receiving attention because they undergo a redox cycle and generate reactive oxygen species (ROS) in target cells [6]. Findings of in vitro experimental studies indicated that ROS generated in response to DEP exposure plays a role in subsequent oxidative stress responses [7,8,9,10,11]. Our previous in vivo studies indicated that antioxidant response elements may determine the host’s susceptibility to the adverse effects of DE [12,13]. The susceptibility to air pollution is determined by gene-environmental interactions. Nuclear factor erythroid-derived 2-like 2 (Nfe2l2), which is also known as NF-E2-related factor 2 (Nrf2), is a transcription factor that is essential for the induction and/or constitutive expression of phase II and antioxidant enzymes [14]. It is reported that oxidative DNA damage and the accumulation of 8-hydroxydeoxyguanosine in the lungs of Nrf2 knockout mice is exaggerated during exposure to diesel exhaust fumes [15]. Nrf2 is a key transcription factor that regulates antioxidant defense in macrophages and epithelial cells that act as the main defense against the proinflammatory and oxidizing effects of DEP [11]. Our previous study suggested that oxidative stress induced by DE is associated with airway inflammation [16], allergic asthma [17], and pulmonary fibrosis [18], as evidenced in experiments using *Nrf2* knockout mice.

We have reported that anti-oxidant responsive elements may determine the host’s susceptibility to low-dose DEP (100 µg/m^3^, which is similar to the level inhaled outdoors near to a road with heavy traffic) in vivo studies [12,13,16,17], may specially contribute to exaggeration of allergic airway inflammatory responses caused by oxidative stress. However, it has also been reported that the hourly high concentration of PM2.5 in some urban environments has a major effect [19]. Some reports have suggested that DE alters immune responses in the lung and may increase susceptibility to pathogens at both high (DEP: 3 mg/m^3^) and low (DEP: 100 µg/m^3^) dose exposure [20,21]; however, the mechanism involved is not clear. This study was designed to confirm the effects of high-dose DE (DEP: 1 mg/m^3^, which is similar to the concentration that is inhaled in urban environments subjected to serious air pollution) in the airway immune response and to identify the molecular mechanisms involved in these effects using *Nrf2*^+/+^ and *Nrf2*^−/−^mice.

## 2. Materials and Methods

### 2.1. Animals

Wild-type (*Nrf2*^+/+^) C57BL/6 mice were purchased from CLEA Japan (Tokyo, Japan). Nrf2 knockout (*Nrf2*^−/−^) C57BL/6 mice were initially obtained from RIKEN BRC (RBRC No. 01390, Tsukuba, Japan) and backcrossed onto the C57BL/6 background in our laboratory. *Nrf2*^−/−^ C57BL/6 mice were generated as described previously [14]. The mice were genotyped for Nrf2 via the PCR-based amplification of genomic DNA extracted from the tail, as described previously [16,17,18]. Briefly, PCR amplification was performed using the following three primers: *Nrf2*-sense for both genotypes: 5′-TGGACGGGACTATTGAAGGCTG-3′; *Nrf2*-antisense for wild-type mice: 5′-GCCGCCTTTTCAGTAGATGGAGG-3′; *Nrf2*-antisense for LacZ: 5′-GCGGATTGACCGTAATGGGATAGG-3′. The amplification conditions involved 30 cycles of 96 °C for 20 s, 59 °C for 30 s, and 72 °C for 45 s. The wild-type allele produced a 734-bp band, whereas the knockout allele produces a 449-bp band. The mice used in this study were 6–8 weeks old and were housed in specific pathogen-free conditions. All procedures were approved by the Animal Care and Use Committee and the Genetic Modification Safety Committee of Nippon Medical School (approval code: 21-7). The mice were randomly classified into each experimental group and housed in wire-mesh cages with clean air or a DE exposure chamber (Nanoparticles Health Science Research Center, Tokyo University of Science, Noda-shi, Japan). All procedures were approved by Tokyo University of Science’s Animal Care and Use Committee.

### 2.2. DE Exposure

The mice were exposed to DE in an inhalation chamber at the Nanoparticles Health Science Research Center, Tokyo University of Science, in accordance with the method described in previous reports [22,23]. Briefly, a 2179 L, 39 Kw/3000 rpm diesel engine (Isuzu Motors Ltd., Tokyo, Japan) was used. The mass and concentrations of DEP were measured using a Piezobalance dust monitor (model 3521; Kanomax Inc., Osaka, Japan) and a condensation particle counter (model 3007; TSI Inc., Shoreview, MN, USA), respectively. The concentrations of gas components (nitric oxide (NOx), sulfur dioxide (SO_2_), and carbon monoxide (CO)) in the chambers were measured using an NO-NO_2_-NOx analyzer (model 42i, Thermo Fisher Scientific Inc., Franklin, MA, USA), an enhanced trace level SO_2_ analyzer (model 43i-TLE, Thermo Fisher Scientific Inc.), and a CO analyzer (model 48i, Thermo Fisher Scientific Inc.). As reported previously [18], the concentration of DEP in DE gas was adjusted to approximately 1 mg/m^3^. The mean concentration of DE is shown in Table 1.

### 2.3. Study Design

Both *Nrf2*^+/+^ and *Nrf2*^−/−^ C57BL/6J mice were used. Mice were exposed to DE (DE group) or to clean air (control group) for 8 h/day and 6 days/week. The mice in all groups were sacrificed by an intraperitoneal injection with an overdose of pentobarbital (Somnolently, Kyoritsu Seiyaku Corporation, Tokyo, Japan) at 4 weeks after DE exposure. After DE exposure for 4 weeks, all groups were examined for the cell populations in bronchoalveolar lavage (BAL) fluid (BALF) from the right lung, and the histopathologic features of the lung tissue through electron microscope images of the left lung. We also measured the concentrations of inflammatory cytokines and chemokines in the BALF or the lung tissues, and the expression of antioxidant mRNA in the lung tissues.

### 2.4. Electron Microscopy Analysis

Electron microscopy was performed in accordance with the method described in previous reports [24], with some minor modifications. Briefly, lung tissues were prefixed in cacodylate-buffered 1% glutaraldehyde and 4% formalin (pH 7.4) for 24 h. at 4 °C, washed in cacodylate buffer, and post fixed with 2% osmium tetroxide (Nisshin EM) for 1 h. After washing in cacodylate buffer, the tissue samples were dehydrated using a graded series of ethanol (up to 100%) and propylene oxide (Nisshin EM), and then embedded in Epon 812 (Nisshin EM). Ultra-thin sections (80-nm thick) were cut using an ultra-microtome MT-XL (RMC, Tucson, AZ, USA). Some sections were double stained with uranyl acetate and lead citrate. They were then observed using a transmission electron microscope (TEM; JEM-1400; JEOL, Tokyo, Japan) with an accelerating voltage of 80–90 kV.

### 2.5. BAL and Cell Count in BALF

BALF was obtained by injection of 0.5 mL of saline solution (three times; total, 1.5 mL) followed by gentle aspiration of the fluid from the right lung after securing an intratracheal catheter within a main bronchus. With this catheter, recovery rates of lavage fluids were 87.0 ± 3.9% and did not differ significantly between groups. Cell counts in BALF were determined as described previously [16]. Briefly, the total number of cells in the BALF was counted using a hemocytometer. To obtain the BALF differential cell counts, Cytospin (Thermo Fisher Scientific, Yokohama, Japan) smear slides were prepared. The cell counts were obtained using standard light microscopy and staining with May-Giemsa (Diff-Quik, Sysmex, Kobe, Japan). Differential cell counts were performed on 200 cells per smear.

### 2.6. Quantitation of Cytokine Protein Levels in BALF

The concentration of macrophage inflammatory protein (MIP)-2, surfactant protein (SP)-D, tumor necrosis factor (TNF)-α, granulocyte macrophage colony-stimulating factor (GM-CSF), interleukin (IL)-17, and IL-33 in the BALF was determined using an enzyme-linked immune sorbent assay (ELISA) kit in accordance with the manufacturer’s instructions (R&D Systems, Minneapolis, MN, USA) respectively.

### 2.7. Quantitation of Cytokine Protein Levels in Lung Tissue Supernatants

Each lung tissue specimen was homogenized using cell lysis buffer. The cell lysis buffer consisting of 50 mM Tris-HCl pH 7.2, 0.5 mM EDTA, 0.15 M NaCl, and 0.5% NP-40. The homogenates were then centrifuged at 100,000× *g* for 1 h. Total protein concentrations in the supernatants were measured using the Bio-Rad Protein Assay to normalize the concentration of target cytokines in the supernatants. In the manner described above for BALF, tissue supernatant was assayed for the protein levels of TNF-α, GM-CSF, IL-17, and IL-33 (R&D Systems, Minneapolis, MN, USA).

### 2.8. Quantitative Real-Time Reverse Transcription-Polymerase Chain Reaction

Extraction of the total RNA and real-time reverse transcription-polymerase chain reaction (RT-PCR) were performed as described previously [18]. Briefly, total RNA was extracted from each lung tissue specimen using ISOGEN (Nippon Gene, Tokyo, Japan) in accordance with the manufacturer’s instructions. Complementary DNA (cDNA) was synthesized using a kit (High-Capacity cDNA Reverse Transcription Kit with RNase inhibitor; Applied Biosystems, Foster City, CA, USA) and quantified with a sequence detector (7500/7500 Fast Real-Time PCR System; Applied Biosystems) using TaqMan Universal PCR Master Mix (Applied Biosystems) and the relevant primers (Applied Biosystems), including a β-actin control. The mRNA expression levels of all samples were normalized to the level of the housekeeping gene β-actin. The names of the target genes and their assay IDs were as follows: β-actin: Mm00607939_s1; Heme oxygenase (HO)-1: Mm00516005_m1; NAD(P)H quinone dehydrogenase (NQO)1: Mm01253561_m1.

### 2.9. Statistical Analysis

Data are expressed as mean ± standard deviation (SD) values. The data were analyzed for significance using one-way ANOVA followed by Newman–Keuls test to adjust for multiple comparisons (Stat Mate III software package; ATMS Digitals Medical Station, Tokyo, Japan). Student’s *t*-test was used to determine the significance of differences between paired comparisons. Probability values of less than 0.05 were considered significant.

## 3. Results

### 3.1. Differential Cell Counts in BALF

**Figure 1:**
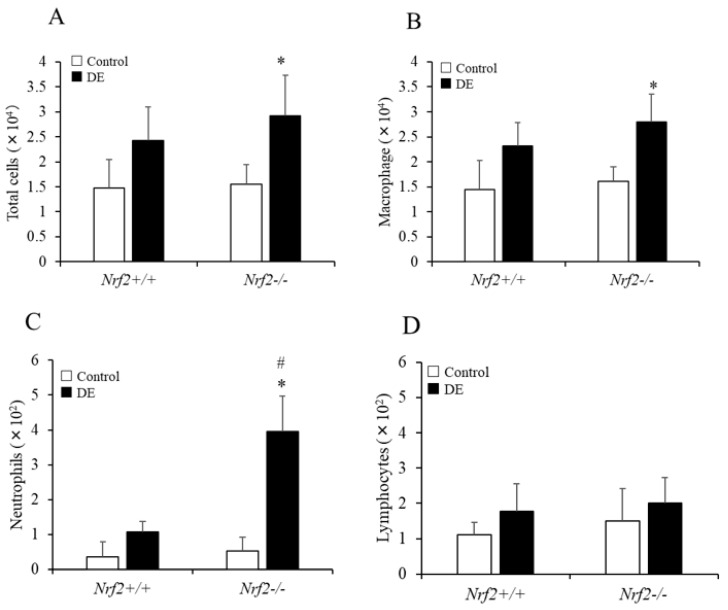
Differential cell counts in bronchoalveolar lavage fluid (BALF). (**A**) total cells, (**B**) macrophages, (**C**) neutrophils, (**D**) lymphocytes. Data are shown as mean ± SD values in each group (*Nrf2*^+/+^ control: *n* = 6; *Nrf2*^+/+^ DE: *n* = 6; *Nrf2*^−/−^ control: *n* = 4; *Nrf2*^−/−^ DE: *n* =5), * *p* < 0.05 vs. control; # *p* < 0.05 vs. *Nrf2*^+/+^.

The numbers of total cells, macrophages, and neutrophils in the BALF increased after DE exposure in both *Nrf2*^+/+^ mice and *Nrf2*^−/−^ mice compared with the control group, respectively; however, the increases induced by DE were significant in *Nrf2*^−/−^ mice, and there were not observed significant changes in *Nrf2*^+/+^ mice (Figure 1A–C). The number of neutrophils in the BALF after DE exposure was significantly higher in *Nrf2*^−/−^ mice than in *Nrf2*^+/+^ mice (Figure 1C). The lymphocytes count induced by DE also was slightly increased in both of *Nrf2*^+/+^ and *Nrf2*^−/−^ mice compared with the control group, respectively; however, there were no significant differences (Figure 1D).

### 3.2. DEP-Laden Alveolar Macrophages in BALF

**Figure 2:**
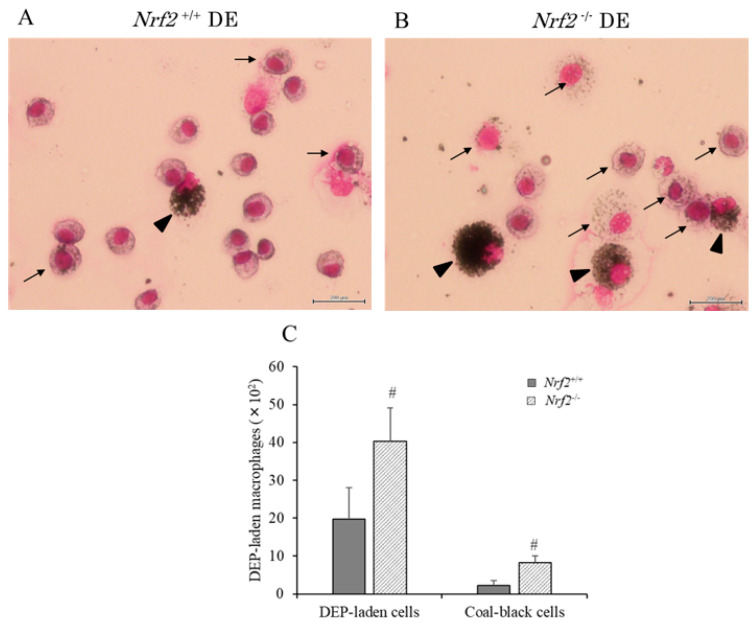
The pathologic features of diesel exhaust (DE) particles (DEP)-laden alveolar macrophages in BALF. (**A**) DE group in *Nrf2*^+/+^ mice; (**B**) DE group in *Nrf2*^−/−^ mice. Representative optical micrographs of May–Giemsa (Kobe, Japan) stained cell preparations. Scale bar was 200 μm. Arrows indicate DEP-laden alveolar macrophages. DEP-laden macrophages in which DEP accounted for more than half of the cytoplasm were defined as coal-black cells. Arrowhead indicates coal-black alveolar macrophages. (**C**) Percentages of DEP-laden macrophages and coal-black alveolar macrophages in total macrophages. Data are shown as mean ± SD values in each group (*Nrf2*^+/+^ DE: *n* = 6; *Nrf2*^−/−^ DE: *n* = 5), # *p* < 0.05 vs. *Nrf2*^+/+^.

We assessed DEP-laden alveolar macrophages in BALF of the DE exposure group using optical micrographs. Typical pictures of DEP-laden alveolar macrophages (arrows) are shown in Figure 2A,B. It was evident that alveolar macrophages engulfed DEP in both the *Nrf*2^+/+^ and *Nrf2*^−/−^ mice in the DE groups. However, the DEP content of each macrophage was more in *Nrf2*^−/−^ mice (Figure 2B) than in *Nrf2*^+/+^ mice (Figure 2A). The DEP-laden alveolar macrophage counts were performed in ten separate fields per smear. The number of DEP-laden alveolar macrophages was significantly higher in *Nrf2*^−/−^ mice than in *Nrf2*^+/+^ mice. The number of coal-black alveolar macrophages, in which DEP accounted for more than half of the cytoplasm, such as those indicated in Figure 2A,B (arrowhead) was also significantly higher in *Nrf2*^−/−^ mice than in *Nrf2*^+/+^ mice (Figure 2C).

### 3.3. Electron Microscopic Analysis

**Figure 3:**
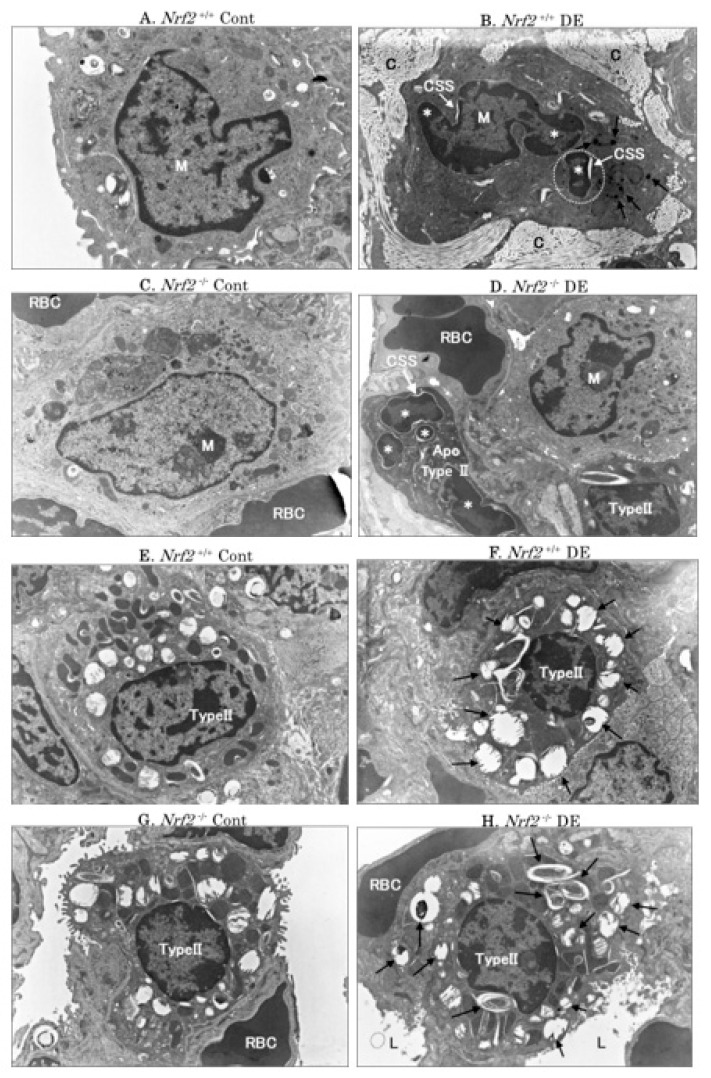
Electron microscopic analysis of alveolar macrophages and alveolar type II epithelial cells (Type II cells). The photographs show representative results. Alveolar macrophages in *Nrf2*^+/+^ mice, control group (**A**), DE group (**B**); alveolar macrophages in *Nrf2*^−/−^ mice, control group (**C**), DE group (**D**). Type II cells in *Nrf2*^+/+^ mice, control group (**E**), DE group (F); Type II cells in *Nrf2*^−/−^ mice, control group (**G**), DE group (**D**,**H**). Black arrows indicate alveolar macrophages engulfing DEP (**B**), and degeneration of the lamellar body (**F**,**H**). A white dotted ring indicates the apoptotic body that was phagocytosed by a macrophage (**B**). M: macrophage; Type II: alveolar type II epithelial cells; *: irregular condensed chromatin and nuclear fragmentation; CSS: crescent-shaped spaces (appearing around the nucleus of an apoptotic cell); Apo: apoptosis; RBC: red blood cell; C: collagen fiber; L: lumen.

Representative pictures of alveolar macrophages and alveolar type II epithelial cells obtained using an electron microscope are shown in Figure 3. Alveolar macrophage-engulfed DEP (black arrows) and apoptotic bodies (marked with a white dotted ring) were observed by electron microscopy in the *Nrf2*^+/+^ mice DE group; the apoptotic bodies comprises irregular-condensed chromatin, nuclear fragmentation (asterisk) and crescent-shaped spaces (CSS) (Figure 3B). Apoptotic bodies appearing around the nucleus of apoptotic cells were also observed in macrophages in the *Nrf2*^+/+^ mice DE group (Figure 3B) and the alveolar type II epithelial cells (Type II cells) in the *Nrf2*^−/−^ mice DE group (Figure 3D). The degeneration of lamellar bodies (black arrows) in Type II cells was observed in the DE groups, especially in *Nrf2*^−/−^ mice (Figure 3H) with severe degeneration (Figure 3F).

### 3.4. MIP-2 and SP-D Levels in BALF

**Figure 4:**
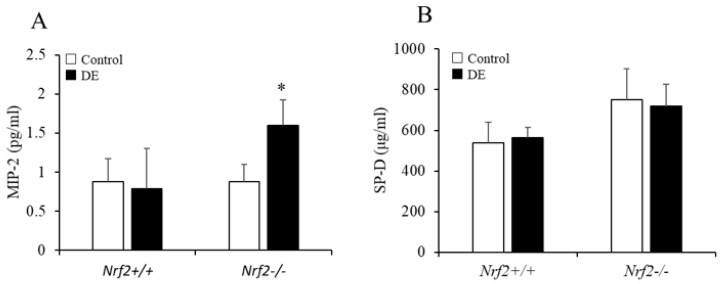
Macrophage inflammatory protein (MIP)-2 (**A**) and surfactant protein (SP)-D (**B**) in BALF were analyzed using ELISA. The vertical axis shows percent changes in the cytokines of the DE group relative to the control group. Data are shown as mean ± SD values in each group (*n* = 5). * *p* < 0.05 vs. control.

The concentration of MIP-2 in BALF significantly increased after DE exposure in *Nrf2*^−/−^ mice; however, there were no significant changes after DE exposure in *Nrf2*^+/+^ mice (Figure 4A). Concerning the concentration of surfactant protein (SP)-D in BALF, no change was found after DE exposure in either *Nrf2*^−/−^ mice, or *Nrf2*^+/+^ mice (Figure 4B). TNF-α, GM-CSF, IL-17, and IL-33 were not detected in BALF.

### 3.5. TNF-α, GM-CSF, IL-17, and IL-33 Levels in Lung Tissue

**Figure 5:**
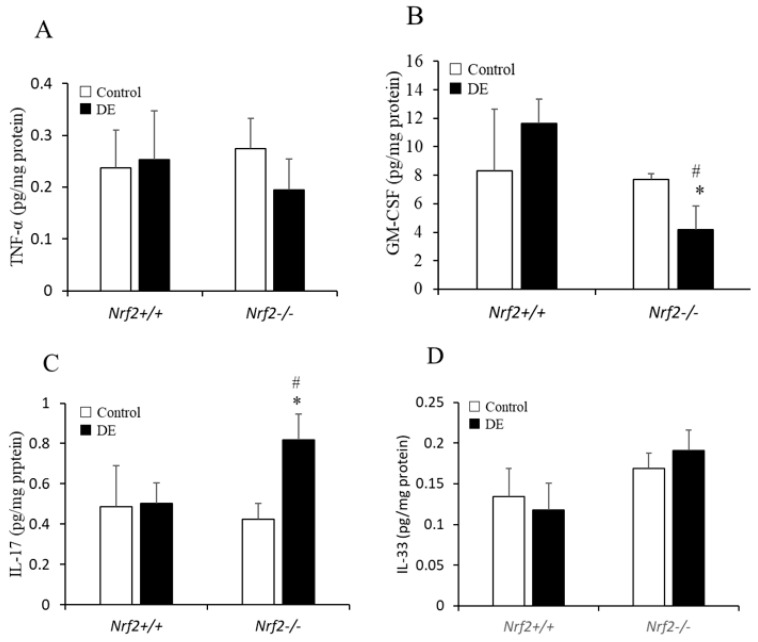
TNF-α (**A**), GM-CSF (**B**), IL-17 (**C**), and IL-33 (**D**) levels in lung tissue supernatants were analyzed using ELISA. Total protein concentrations in the supernatants were measured to normalize the concentration levels of target cytokines in the supernatants. The vertical axis shows percent changes in the cytokines of the DE group relative to the control group. Data are shown as mean ± SD values in each group (*n* = 5). * *p* < 0.05 vs. control; # *p* < 0.05 vs. *Nrf2*^+/+^.

TNF-α, GM-CSF, IL-17, and IL-33 levels in BALF were not detected using ELISA; therefore, these cytokine levels in lung tissue supernatants were detected using ELISA. The concentration of TNF-α in lung tissue was slightly decreased after DE exposure in *Nrf2*^−/−^ mice; however, there was no significant change found compared with control group (Figure 5A). The concentration of GM-CSF in lung tissue was significantly decreased after DE exposure in *Nrf2*^−/−^ mice, and was lower in *Nrf2*^−/−^ mice than in *Nrf2*^+/+^ mice (Figure 5B). The concentration of IL-17 in lung tissue was significantly increased after DE exposure in *Nrf2*^−/−^ mice, and was higher in *Nrf2*^−/−^ mice than in *Nrf2*^+/+^ mice (Figure 5C). Concerning the concentration of IL-33 in lung tissue, it was slightly increased after DE exposure in *Nrf2*^−/−^ mice compared with control group; however, there were no significant differences (Figure 5D).

### 3.6. Induction of Antioxidant Enzyme mRNA Expression in the Lung Tissues

**Figure 6:**
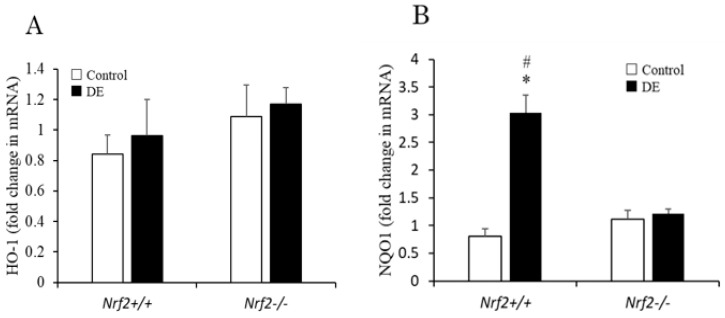
HO-1 (**A**) and NQO1 (**B**) mRNA expression levels in the lung tissues were analyzed using real time RT-PCR. The vertical axis shows percent changes in target gene mRNA expression levels of the DE group relative to the control group. β-actin was used as an internal control. Data are shown as mean ± SD values in each group (*n* = 3). * *p* < 0.05 vs. control; # *p* < 0.05 vs. *Nrf2*^−/−^.

The mRNA expression levels of HO-1 and NQO1 in the lung tissue were determined by quantitative RT-PCR reaction. Concerning the mRNA expression level of HO-1 in lung tissue, no remarkable change was found after DE exposure in either *Nrf2*^−/−^ mice, or *Nrf2*^+/+^ mice (Figure 6A). The mRNA expression level of NQO1 in the lung tissue was significantly increased in *Nrf2*^+/+^ mice compared with control group, and higher in *Nrf2*^+/+^ mice than in *Nrf2*^−/−^ mice (Figure 6B).

## 4. Discussion

**Figure 7:**
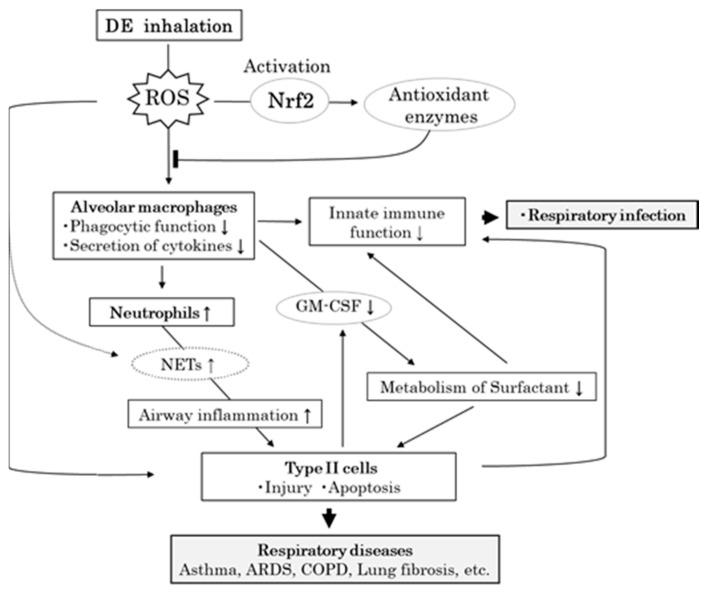
Schematic diagram of putative mechanism of oxidative stress and its effect on airway immune response to DE exposure in mice. DE: diesel exhaust; ROS: reactive oxygen species; NETs: neutrophil extracellular traps; GM-CSF: granulocyte macrophage colony-stimulating factor; Type II cells: alveolar type II epithelial cells; COPD: chronic obstructive pulmonary disease; ARDS: acute respiratory distress syndrome; Solid line: putative signaling pathways in the airway response to DEP exposure; Dashed line: unclear signaling pathways; Arrowhead: promoted in *Nrf2*^−/−^; ↓: inhibited in *Nrf2*^−/−^; ↑: stimulated in *Nrf2*^−/−^.

This study was designed to confirm the effects of DE (DEP: 1 mg/m^3^, which is similar to the concentrations that are inhaled in urban environments with serious air pollution) to identify the molecular mechanisms involved in these effects and in the development of DE-induced airway immune responses using *Nrf2*^+/+^ and *Nrf2*^−/−^ mice. The results indicated that inhalation of DE was associated with greater inhibition of alveolar macrophage function and increase in neutrophil numbers in *Nrf2*^−/−^ mice than in *Nrf2*^+/+^ mice. Alveolar type II cell injury was also remarkably induced in *Nrf2*^−/−^ mice, as observed using an electron microscope. Our results suggested that Nrf2 reduces the risk for pulmonary diseases via modulating the airway innate immune response induced by DE in mice (Figure 7).

Alveolar macrophages are 15–50 μm in diameter, they are mainly located in the alveolar space, and they represent the predominant phagocytic and antigen-presenting cell in the human respiratory tract [25]. Normally, alveolar macrophages are the most abundant cellular fraction within BALF, whereas under inflammatory conditions other leukocyte populations, prototypical neutrophils and lymphocytes accumulate and shift this balance. Alveolar macrophages are the sentinel phagocytic cell of the innate immune system in the lungs [26]. Fine particles such as DEP are carried to the alveolar surface where they interact with soluble components in alveolar fluids and alveolar macrophages. Both the organic and particulate components play a role in DEP-induced pulmonary toxicity [27]. Previous studies showed that DEP, through their organic component, impair the phagocytic, bactericidal, and secretory function of alveolar macrophages, leading to increased susceptibility of the lung to bacterial infection [28,29].

In the present study, the DEP content of alveolar macrophages in BALF were remarkably higher in *Nrf2*^−/−^ mice than in *Nrf2*^+/+^ mice, which suggests that Nrf2 is involved with the alteration of airway clearance function by DE exposure. TNF-α is produced chiefly by activated macrophages as an important pro-inflammatory cytokine. Remarkable changes in TNF-α expression in the lung tissue were not observed in *Nrf2*^+/+^ mice, and mild suppression of TNF-α expression in *Nrf2*^−/−^mice was seen after DE exposure in this study. A recent study suggests that biodiesel (DB) particulate matter by intranasal instillation promotes oxidative stress by activating the Nrf2/HO-1 and inflammation by p-NF-kB/TNF-α pathways. The protein expression levels of Nrf2, p-NF-kB, and HO-1 were higher in the low-dose DB (250 μg) group and lower in the high-dose DB (1000 μg) group than in control mice and the low-dose DB group [30]. It also has been reported that the TNF-α secretion by alveolar macrophages was reduced after high-dose DE exposure (3 mg/m^3^) in mice [20]. Therefore, it is conceivable that activating the Nrf2 pathway and inflammation by TNF-α to DE exposure was related to the oxidative stress levels such as DE exposure concentration. It may also explain the lack of an increase in the level of TNF-α in the lung tissue in *Nrf2*^+/+^ mice and mild suppression of the level of TNF-α in *Nrf2*^−/−^ mice after exposed DE (DEP 1 mg/m^3^) in the present study.

GM-CSF is expressed in a variety of hematopoietic cell types and nonhematopoietic cells, such as macrophages, mast cells, epithelial cells, etc. [31], and it regulates alveolar macrophage phagocytosis between innate and adaptive immunity in the lung [32]. GM-CSF expression in the lung tissues was slightly increased in *Nrf2*^+/+^ mice and significantly decreased in *Nrf2*^−/−^mice after DE exposure in this study. The lack of an increase in the level of TNF-α in the lung tissue and the significant suppression in the level of GM-CSF in lung tissue in *Nrf2*^−/−^mice after DE exposure, suggests that the proinflammatory cytokine secretion function in macrophages is also attenuated by the DE exposure-mediated Nrf2 pathway. This would be consistent with the observation that the activation of alveolar macrophage antioxidant defenses is mediated through Nrf2 and its downstream effectors [33]. The functions of alveolar macrophages being attenuated by the DE exposure-mediated Nrf2 pathway were also consistent with a previous study in a murine bleomycin lung fibrosis model [18]. Cytoprotective pathways are induced by the Nrf2 transcription signal pathway at the lowest levels of oxidative stress, and this may constitute the first tier of a hierarchical oxidative stress response, as is in the production of antioxidant enzymes. If these enzymes fail to neutralize the effects of ROS, proinflammatory effects constitute a second tier or superimposed level of oxidative stress. The final tier or superimposed level of oxidative stress is cytotoxicity, including the initiation of programmed cell death [9]. Nrf2 is a key transcription factor that regulates antioxidant defense in macrophages and epithelial cells that constitute as a main defense action against the proinflammatory and oxidizing effects of DEPs [11]. The lack of an increase in the level of TNF-α and significant suppression in the level of GM-CSF after DE exposure in *Nrf2*^−/−^mice in this study may be associated with cytotoxicity, including the initiation of programmed cell death. DE inhalation-induced apoptotic bodies [34] and CSS, which appear around the nucleus of apoptotic cells [35], were also observed in macrophages that may be associated with macrophage hypofunction following DE exposure, while excess oxidative stress further induced the macrophage dysfunction in *Nrf2*^−/−^mice. These observations suggest that DE alters innate immune function in the lung and Nrf2 reduces the risk of lung infections.

Suppression of macrophage function also affects antigen presentation to lymphocytes; therefore, lymphocytes do not respond, and this promotes neutrophil recruitment to DE exposure in both *Nrf2*^+/+^ and *Nrf2*^−/−^ mice, and number of neutrophils in the BALF was significantly higher in *Nrf2*^−/−^ mice compared with *Nrf2*^+/+^ mice. Human challenge studies with DE have also revealed an increase in airway neutrophilic inflammation [36,37]. MIP2 is a C-X-C motif chemokine 2 (CXCL2), and it has been shown to stimulate the migration and activation of neutrophils [38]. The cytokine IL-17 is a major orchestrator of sustained neutrophilic mobilization [39]. It has been reported that Th17 differentiation is regulated via Nrf2 pathway [40]. The MIP-2 levels in BALF, and IL-17 levels in the lung tissues, also increased significantly in *Nrf2*^−/−^ mice compared with *Nrf2*^+/+^ mice after DE exposure in this study, suggesting that promotion of neutrophil recruitment to DE exposure in *Nrf2*^−/−^ mice was mediated by MIP2 and IL-17 in the present study. Neutrophil extracellular traps (NETs) are released by neutrophils and cause local tissue damage and inflammation [41,42]. This active process is dependent on the generation of ROS by NADPH oxidase [43]. Therefore, it is considered that NETs may be involved in airway inflammation caused by oxidative stress from DE exposure, and Nrf2 is speculated to be a protective factor against epithelial cell injury. IL-33 is an IL-1-related cytokine that can act as an alarmin when released from necrotic cells, and it also regulates neutrophil recruitment [44]. The balance between oxidative stress and antioxidant responses plays a key role in controlling IL-33 release in airway epithelium [45]. However, no association between IL-33 and Nrf2 was observed in the neutrophilic airway inflammation caused by DE in the present study.

Surfactant is a complex mixture of lipids and proteins that forms a monolayer lining the alveolar sacs in the lungs to maintain surface tension and prevent the collapse of alveoli [46]. Surfactant is composed of phospholipids, neutral lipids, and proteins. The surfactant proteins (SPs) play critical roles in the formation, function, and metabolism of surfactant [47,48]. SP-A and SP-D also play critical roles in the immune response to foreign antigens [48,49]. Surfactant lipids are synthesized and secreted by alveolar type II cells. Type II cells are the major cells responsible for the turnover of surfactant. Certain lamellar bodies and other vesicles seen within Type II cells may actually be a part of an endocytotic recycling pathway rather than the exocytotic secretory pathway of the surfactant [50]. In the present study, degeneration of the lamellar body in Type II cells were observed in DE groups, and *Nrf2*^−/−^ mice had especially severe degeneration compared with *Nrf2*^+/+^ mice. This finding is considered to be an abnormality in the metabolism of the surfactant. The levels of SP-D in BALF were not changed by DE exposure. It has been reported that SP-A and SP-D are secreted through a different lamellar body-independent pathway [51]. GM-CSF regulates the clearance or catabolism rather than synthesis of surfactant proteins and lipids. It has been reported that respiratory epithelial cells synthesize GM-CSF, which is consistent with the hypothesis that GM-CSF plays an important role in surfactant metabolism [30]. In our study, GM-CSF levels in lung tissues increased markedly after DE exposure in *Nrf2*^+/+^ mice but decreased markedly after DE exposure in *Nrf2*^−/−^ mice. Insufficient production of GM-CSF in lung tissue due to macrophage and Type II cells dysfunction after DE exposure in *Nrf2*^−/−^ mice thus impairs detergent protein clearance or catabolism, resulting in metabolic disorders of surfactants. Electron microscopic findings of lamellar degeneration in Type II cells may reflect metabolic disorders of the surfactant.

In vitro studies have reported that DEP induces apoptosis by oxidative stress [52]. In this experimental system as well, apoptotic findings of Type II cells were observed by electron microscopy after exposure to DE. In particular, *Nrf2*^−/−^ mice suffered severe damage compared with *Nrf2*^+/+^ mice. Further studies are needed on the molecular mechanism that causes DE-induced apoptosis of alveolar epithelial cells in this experimental system.

*Nrf2*^+/+^ mice exhibited significantly higher pulmonary mRNA expression levels of the antioxidant enzyme NQO-1 after DE exposure. This was consistent with a previous report about the effect of DE inhalation (DEP: 1 mg/m^3^) in a bleomycin-induced lung fibrosis model. In the present study, we did not observe significant changes in HO-1 mRNA expression levels in *Nrf2*^+/+^ mice. Our previous report showed that after low-dose DE (DEP: 100 μg/m^3^) exposure for 8 weeks, the respective fold changes in HO-1 mRNA expression levels in the lungs of *Nrf2*^+/+^ and *Nrf2*^−/−^ mice were 11.8 vs. 6.9 [16]. Our previous report also showed that in BALB/c mice, expression of HO-1 mRNA in lung tissues was markedly increased after 1 week of low-dose DE exposure; in C57BL/6 mice, expression of HO-1 mRNA in lung tissues was marked after 4 and 8 weeks of low-dose DE exposure [12]. It is possible that the response of host antioxidant enzymes to DE exposure was related to the animal strain, DE exposure concentration, and exposure time course.

Of note, after low-dose DE (DEP: 100 μg/m^3^) exposure for 8 weeks, Th2-dominated airway inflammation was induced in *Nrf2*^−/−^ C57BL/6 mice [16]. In contrast, in this study, with high-dose DE (DEP: 1 mg/m^3^) exposure for 4 weeks, airway inflammation mainly due to innate immune responses was induced in Nrf2^−/−^ C57BL/6 mice. Nrf2 is a key factor involved in airway inflammation via the modulation of airway innate immune or acquired immune responses induced by oxidative stresses from DE inhalation exposure.

## 5. Conclusions

Our present study suggested that Nrf2 is an important protective factor against lung injury via modulating the airway innate immune response induced by oxidative stresses from DE, and it can lower the risk of some respiratory diseases such as respiratory infection, asthma, acute respiratory distress syndrome, chronic obstructive pulmonary disease, and pulmonary fibrosis.

## Figures and Tables

**Table 1:** The concentration of gases and particles in each chamber.

Chamber	CO (ppm)	SO_2_ (ppb)	NO (ppm)	NO_2_ (ppm)	NO_x_ (ppm)	DEP (mg/m^3^)	DEP (#/cc)
Clean	0.44 ± 0.17	0.64 ± 0.50	0.00 ± 0.01	0.02 ± 0.01	0.02 ± 0.01	0.01 ± 0.01	3 ± 1
DE	10.26 ± 2.72	21.03 ± 5.50	3.65 ± 0.84	1.91 ± 0.45	5.55 ± 1.26	1.02 ± 0.29	343,700 ± 2900

The values are mean ± SD of data in each group (*n* = 40 days). #: number.
